# Striking rise of cesarean section rates in Türkiye: there is a need for a whole new perspective

**DOI:** 10.11604/pamj.2024.48.6.12380

**Published:** 2024-05-03

**Authors:** Mert Küçük

**Affiliations:** 1 Department of Obstetrics and Gynecology, Mugla Sitki Kocman University, Faculty of Medicine, 48000 Menteşe, Muğla, Türkiye,; 2 Department of Medical Education and Bioinformatics, Faculty of Medicine, 48000 Menteşe, Muğla, Türkiye

**Keywords:** Cesarean section rate, Türkiye, Health Transformation Program (HTP)

## Abstract

Since 2003, the Turkish Ministry of Health (TMOH) has activated a reformed system called Health Transformation Program (HTP) which has assertive goals. Health transformation program has brought about important improvements in many health topics. However, at the beginning of HTP, cesarean section (C-section) rate was approximately 30%, having exceeded 50% in 2013 which reflected the highest rate in Organization for Economic Cooperation and Development (OECD). Currently, most of the deliveries are carried out via C-section in Türkiye which started disputes about whether the high rate of C-section is Achilles’ heel of HTP. To overcome high C-section rate, TMOH has been making intensive efforts and taking serious measures in recent years including passing a law to ban elective C-sections. Despite the strict measures taken C-section rate didn’t decrease instead increased gradually. The current situation shows that the problem is more complicated than the authorities figure out, and a whole new perspective on the issue is needed.

## Perspectives

Since 2003, the Turkish Ministry of Health (TMOH) has activated a reform system in health called the Health Transformation Program (HTP) which has ambitious goals. Since then, HTP has brought about important improvements in many health topics [1]. However, at the beginning of HTP, the cesarean section (C-section) rate was approximately 30%, having exceeded 50% in 2013 which was the Organisation for Economic Co-operation and Development (OECD)'s highest. The C-section rate has currently reached 58.4% which is the highest rate globally [2]. Currently, most of the deliveries are carried out via C-section in Türkiye which started disputes about whether the high rate of C-section is Achilles´ heel of HTP.

**C-section rates:** they have escalated dramatically all over the world and it is still rising [3]. This is a common problem encountered in many countries with different cultural, and social backgrounds, medical and technical feasibility [4]. It was reported that the C-section rate was approximately 32.3% in the USA in 2009 and the rate has approximately doubled since 1996 [5]. Nearly one-third of deliveries in USA and Australia are carried out via C-section and this is the highest rate of all time [6]. The C-section rate has increased globally, but even a higher increase in the C-section rate in Türkiye is observed compared to other countries [7]. Unfortunately, with a rate of 51.1%, Türkiye had the highest rate of C-section rate among countries of the OECD in 2014. In 2014, this rate was 25.2% in the United Kingdom, 16.9% in Israel, 16.2% in Holland and 35.7% in Italy.

In addition, there are significant differences in C-section rates among different regions in Türkiye. Turkish Public Hospitals Institution (TPHI) published information about birth rates at public hospitals in provinces. In this report, C-section numbers were figured out among other health statistics at public hospitals in 81 provinces. In this report, huge differences in C-section rates in public hospitals among provinces were observed. For instance, Tunceli, which is a small province, came first in C-section deliveries (74%). The lowest C-section rate belonged to Van and Siirt provinces (22%). Even in these two cities with the lowest rates, the C-section rate was still above the World Health Organization's (WHO) recommended rate.

**Optimal C-section rate discussions:** the most challenging part of identifying the optimal C-section rate is a standardized tool for comparing the different settings or the C-section rate in a population within time. There is a need for a reliable and internationally accepted classification system to produce standard data that can be used as a valuable tool for investigating the trend of C-section rates which are increasing gradually [4]. The classification system should widely be applied with an international consensus to enable the comparison of C-section rate accurately between different regions, countries, and institutions [8]. Even though there are many recommended classifications, 10-group classification system (known as Robson classification) to classify the mode of deliveries is supported by WHO. With the widespread usage of Robson classification, it is possible to compare the rates belonging to different regions and changes in time with more accurate statistics [9]. For nearly 30 years, international health authorities have considered the ideal C-section rate as 10-15%. This rate was first expressed by reproductive health experts in a panel held by WHO in Fortaleza, Brazil [8]. However, there are some important points to note. The announced rate was based on limited clinical data. The data used to identify the optimal C-section rate rather belonged to Northern Europe [8].

Through a systematic analysis with limited data conducted by WHO, it was seen that maternal, newborn, and infant mortality numbers decreased in a society with 10-15% C-section rates. The increase in cesarean deliveries above this level was not found to be associated with a decrease in mortality [8,9] even though there are conflicting studies. One argument about the optimal C-section rate is that in most countries it is much higher than 15% which was recommended by WHO. Organization for economic cooperation and development average even was also higher [2]. This situation brought out the objections whether WHO´s target was utopian.

Turkish Society of Obstetrics and Gynecology (TSOG) stated in their cesarean report that WHO´s 15% target for the C-section rate which belonged to just one publication nearly 30 years ago was not realistic. Therefore, it was stated that the high C-section rate should be decreased to a reasonable level as a first step in Türkiye. According to TSOG, the target should be 35% for Türkiye in the first stage. Currently, reducing the 58.4% rate to 15% is not realistic, especially in a clinical atmosphere where most of the cesarean deliveries are repeat C-sections. In today´s circumstances, determining an unrealistic rate and preparing accordingly may lead to more deviations from the target contrarily.

The discussions emerge some questions that are not easy to answer. Should the optimal rate of C-sections be the same for each country? In Türkiye, where the repeat C-section rate already exceeded 15% in some regions, is WHO´s 15% rate realistic? Should discussions be directed basically to reducing the C-section rate or should be based on the primary C-section rate or another parameter? In countries where elective C-section is considered as a right, how will the optimal C-section rate be determined in case the majority of women want to have cesarean deliveries more than the rate set by WHO? Is the C-section rate recommended supposed to change with time or not? Is the optimal C-section rate supposed to be the same in various regions or not? There are ongoing debates about the optimal C-section rate. These discussions should take part on a larger dimension by taking each country´s clinical, cultural, and psychological factors in addition to the public health data and economics into consideration [4]. I think health authorities in Türkiye are over focusing on optimal C-section rate and this creates some problems. In fact, what needs to be focused on is not to perform C-section to any women except the ones who really needs it. Unfortunately, this reality is overshadowed by the ambitious targets of health authorities to achieve the optimal C-section rate which is 10-15% and strict measures taken to achieve this goal creates other complex problems.

**Hospital types in Türkiye and C-section rates:** health transformation program by TMOH has been in practice since 2003. Since then, number of private hospitals has exploded. In 1998, there were 125 private hospitals and in December 2015, this number reached to 560 compared with 874 public hospitals and 70 university hospitals [10]. According to the 2014 data, when C-section rates were analyzed depending on the type of institution, the C-section rate was 35.5% at public hospitals, 63.8% at university hospitals while it was 69.5% at private hospitals. When primary C-section rates were analyzed, the primary C-section rate at private hospitals was approximately 2.5 times the rate at public hospitals [11]. The C-section rate at public hospitals was half that of the private hospitals. The C-section rate was so dramatically high at private hospitals (69.5%) that no medically justifiable reason could explain this case. Some private hospitals approached the C-section rate of 100% and this made the situation even worse. HTP must surely confront the high primary and total C-section rates at private hospitals in order to decrease the C-section rates. Private sector continues to get more shares from the total health system and if this trend continues, the C-section rate will even increase more. University hospitals are referral centers where high-risk patients are delivered. Because share of the university hospitals in total health care is low and due to high-risk patients referred, the current high rate in university settings may rather be acceptable. However, it should still be examined closely and thoroughly.

**Risks and complications of C-section:** a C-section rate above 10-15% did not lead to any improvement in maternal and fetal morbidity and mortality. On the contrary, the high C-section rate has been associated with increased maternal-fetal morbidity and mortality [3] even though there are studies with conflicting results [12]. Cesarean births were found to be associated with ureteral, vesical injury, placenta previa, and uterine rupture in subsequent pregnancies [13]. In Liu *et al*. study, C-section has been associated with an increased risk of hemorrhage requiring hysterectomy, as well as with venous thromboembolism, postpartum risks of cardiac arrest, major puerperal infection, anesthetic complications, wound hematoma and longer stay in hospital [14]. In the study of Kennare *et al*. the cesarean delivery was found to increase the risks for placenta accreta, prolonged labor, low birth weight, placenta previa, antepartum hemorrhage, emergency C-section, uterine rupture, malpresentation, preterm birth, small for gestational age, stillbirth [15]. Meanwhile, to give an example; in a region in southern Brazil, the C-section rate increased from 28% to 43% between 1982 and 2004. The preterm birth rate also increased from 6% to 16% [16]. Some other causes may also be taken into consideration, one factor may be iatrogenic prematurity related to early C-section timing.

In the study of Getahun *et al*., cesarean deliveries have been associated with an increased risk of previa and abruption in the second trimester. A dose-response pattern was revealed between the risk of previa and increasing number of prior C-sections. Increased risks of previa and abruption were also found to be related to a short interpregnancy interval [17]. In another study, children born by C-section were approximately 20% more likely to be diagnosed with autism spectrum disorder. However, when sibling controls were assessed, the relation did not persist, suggesting that this relation was due to familial confounding by environmental and/or genetic factors [18]. When trying to place an epidural catheter for labor analgesia unintentional dural puncture (UDP) and postdural puncture headache may occur. While headache is a well-known problem in the acute period after UDP, chronic headache and backache are observed as a complication following long after UDP [19]. It has been supposed that medically unnecessary C-sections have brought a serious burden to the health care system. It was calculated that in 2001 with 27.7% C-section rate, which was much lower than today´s 58.4% rate, brought about a $14 million extra charge to the Turkish healthcare system [10]. C-section deliveries were also found to be associated with many different unwanted clinical situations that were not mentioned in the text.

**Measures taken by Turkish health authorities:** in order to ensure the unity of practice in Türkiye and guide the physicians in their clinical practice, the ’Labor Management Guide' was published in 2007. With the cooperation of the Labor and C-section Program Science Commission, TSOG, Turkish Perinatology Society, Maternal-Fetal Medicine, and Perinatology Society of Türkiye, the revised Labor and C-section Management Guideline was published by TMOH in 2010. An intensive campaign is held by TMOH to increase awareness about the risks of cesarean birth in society among the women at childbearing age. In June 2016, Mr. Akdağ, who was the Minister of Health, announced “It is a crime against humanity to prevent the natural birth of a child via C-section while there is no need” and he showed a strong counterposition against rising C-section rate [20].

Another strategy planned by the TMOH was to give a certificate of appreciation to the hospitals that reduced the cesarean delivery rate. Turkish Ministry of Health also supports vaginal birth after C-section (VBAC). Among recommendations included in the Labor and C-section Management Guideline, there is an item about VBAC: “In appropriate cases, vaginal delivery may be recommended after C-section. Risks of attempt should be disclosed to the mother candidate with a detailed patient consent form.” Robson classification which was proposed by WHO as a standard for evaluation of C-section deliveries [8] which enables monitoring and comparing C-section rates has been put into active use in all hospitals by TMOH.

On July 4, 2012, the Turkish parliament passed a new law, to limit births by C-section to cases of medical necessity. Türkiye has been claimed to be the first country to make elective C-sections punishable by law, resulting in fines for doctors who perform elective C-sections. It is stated in the Public Health Act´s article 153 that “only if there is a medical necessity for the pregnant woman and baby in the womb” C-section can be performed. In the process of passing this law and afterward, there are ongoing feminist ethics perspective debates in front of the public about whether it is the right of the mother to decide the type of delivery and whether it is appropriate to intervene. Without distinction of any public, private, or university hospitals, primary C-section rates at all hospitals in Türkiye are subject to regular assessments by TMOH. Hospitals with high primary C-section rates are being closely reassessed again and warned.

It had taken part in the press that in case there was no decline in C-section deliveries TMOH was planning to bring an upper limit for C-sections and for the private hospitals that had C-section rates above the determined limit, fees related to unnecessary C-sections would not be paid via Social Security Institution (SSI). The cancellation of the contracts of these hospitals with SSI would be brought to the agenda. It also took place in the press that doctors whose C-section rates were high would be warned. To the best of the author´s knowledge these regulations had not entered into force. It has taken part in the press that TMOH planned to take four important steps to promote vaginal birth and to reduce the C-section rate. Accordingly, it was brought to the agenda that physicians in obstetrics and gynecology without performing a preset number of normal and difficult births would not be able to acquire an “expertise” certificate following the decisions taken by the Board of Medical Specialties affiliated with TMOH. Turkish Ministry of Health was planning to increase payments for vaginal deliveries by up to 30-40% to the hospitals compared with C-sections and it was aimed to encourage the hospitals to stop the medically unnecessary C-sections. The staff who performed vaginal birth was planned to be paid more and the staff who performed C-sections less according to the new system. Turkish Ministry of Health was supposed to think that obstetricians who faced high medico-legal risks chose C-sections because of the high medical malpractice compensations assumed to be related to normal births and for that reason, it was stated that insurance mandatory for normal deliveries would be put into action at hospitals. The medical malpractice compensations related to vaginal delivery would be paid from the insurance and insurance fees were expected to be paid by hospitals. To the best of author's knowledge, these regulations had not entered into force.

Additionally, TMOH detected that some mother candidates tend to give birth via C-section because of different concerns which is why TMOH organizes awareness campaigns. Within this scope, TMOH has organized pregnancy schools to increase the knowledge about pregnancy, explain labor and birth, ensure the elimination of labor pain fear, inform about natural childbirth, infant care, nutrition, and health, inform pregnant women about the hospital environment, prepare them for the delivery and cope with prenatal and postnatal experienced anxiety. Participants are given a certificate for completing the program. Low activity level and high BMI levels were found to be positively correlated with C-section rates [21]. Of adult females 30.90% are overweight and 23.60% are obese in Türkiye. This is an alarming statistic and TMOH is starting to take steps to reduce obesity in Türkiye [22].

**Are the precautions effective:** despite all the intense organized studies of TMOH since 2008, even despite passing a law special to Türkiye to ban the C-sections performed on maternal request, the C-section rate did not decrease in stead deliveries with C-sections continued to increase. Looking at recent history; in 1988, the C-section rate was 5.7% in Türkiye. In 1993, it was 7%; in 1998, 14%; in 2006, 31.7%; in 2010, 47.2%; in 2014, 51%; in 2021, 58.4%. This rate represented the highest one worldwide. Unfortunately, despite the strict measures taken and efforts to increase awareness, the C-section rate increased steadily [2].

## Conclusion

C-section rates are increasing globally. However, more than half of the births in Türkiye are performed via C-section and this rate is the highest in the OECD. Health authorities in Türkiye have had to take serious measures even including passing a law to ban the C-sections performed on maternal request. Unfortunately, despite the strict measures taken and intense efforts, the C-section rate did not decrease but increased steadily and became the world´s highest. Instead of over-focusing on the optimal C-section rate, TMOH needs to focus on different strategies.

Identification of the main reasons behind the increasing C-section rate is important in order to take effective measures. Scientific studies on this issue should be supported. Taking the views of health staff in the field and acting accordingly is important in this context. Particularly, there is a need for high-quality studies examining the perspective of obstetricians on the increasing C-section rate and these studies have the potential to play a key role in the solution of the problem. As an example, a previously conducted study revealed that the greatest concern among obstetricians who perform cesarean deliveries was malpractice litigation and related high compensation costs. The responses of the participants highlighted that they did not believe that negative reinforcement by government bodies would work to decrease high C-section rates [23]. Another study investigating defensive medicine practices among obstetricians found that a high C-section rate was found to be related to medicolegal concerns including a high litigation atmosphere, low compulsory medical malpractice insurance coverage, and absence of a specific malpractice law in obstetricians´ perspective [24]. TMOH must also confront the problem of high total and high primary C-section rates at private hospitals to decrease the C-section rate. Despite all the measures including passing strict legislation that are taken and hoped to reduce the C-section rate, the rate is increasing gradually. This situation shows that the problem is much more complex than health authorities figure out and there is a need for a whole new perspective on the issue.
